# The tumour microenvironment in paediatric rhabdomyosarcomas: a systematic review

**DOI:** 10.1093/carcin/bgag011

**Published:** 2026-02-20

**Authors:** Megan Richards, Christina Putnam, Timothy J Underwood, Zoë S Walters

**Affiliations:** School of Cancer Sciences, Faculty of Medicine, University of Southampton, Southampton SO17 1BJ, United Kingdom; School of Cancer Sciences, Faculty of Medicine, University of Southampton, Southampton SO17 1BJ, United Kingdom; School of Cancer Sciences, Faculty of Medicine, University of Southampton, Southampton SO17 1BJ, United Kingdom; School of Cancer Sciences, Faculty of Medicine, University of Southampton, Southampton SO17 1BJ, United Kingdom

**Keywords:** Rhabdomyosarcoma, tumour microenvironment, *PAX-FOXO1* fusion gene, immune microenvironment, extracellular matrix

## Abstract

Rhabdomyosarcoma (RMS) is a predominantly paediatric cancer that is classified by the presence or absence of a *PAX-FOXO1* fusion gene, which is associated with a worse prognosis. Previous classification was based on histology, alveolar RMS (ARMS) or embryonal RMS (ERMS). In other paediatric cancers, fusion gene status has been shown to associate with differences in the tumour microenvironment (TME). However, comprehensive understanding of the TME in RMS and how it may differ between subtypes is lacking. This systematic review aimed to identify differences in the TME between fusion-positive RMS and fusion-negative RMS, to better understand how the fusion gene drives malignancy. The Web of Science, MEDLINE (Ovid), and EMBASE (Ovid) were searched to identify relevant studies investigating the TME in RMS. A total of 17 studies met the inclusion criteria and were included in the review, but only three studies specified fusion status in their sample data. Nine studies investigated the extracellular matrix and stroma, and another nine investigated the immune microenvironment. Significant differences in CD163+ macrophages, matrix metalloproteinases and stromal platelet-derived growth factor receptors-α/β were observed between ARMS and ERMS. Regarding fusion status, there were differences in the prevalence of T cell dysfunction, NECTIN-3 expression, and genes related to PD-1 signalling and interferon (IFN) response. This review highlights a need for further research of the TME in each fusion subtype. This will improve our understanding of how the fusion gene drives malignancy and ultimately aids in the development of novel treatment strategies.

## Graphical Abstract

**Graphical Abstract:**
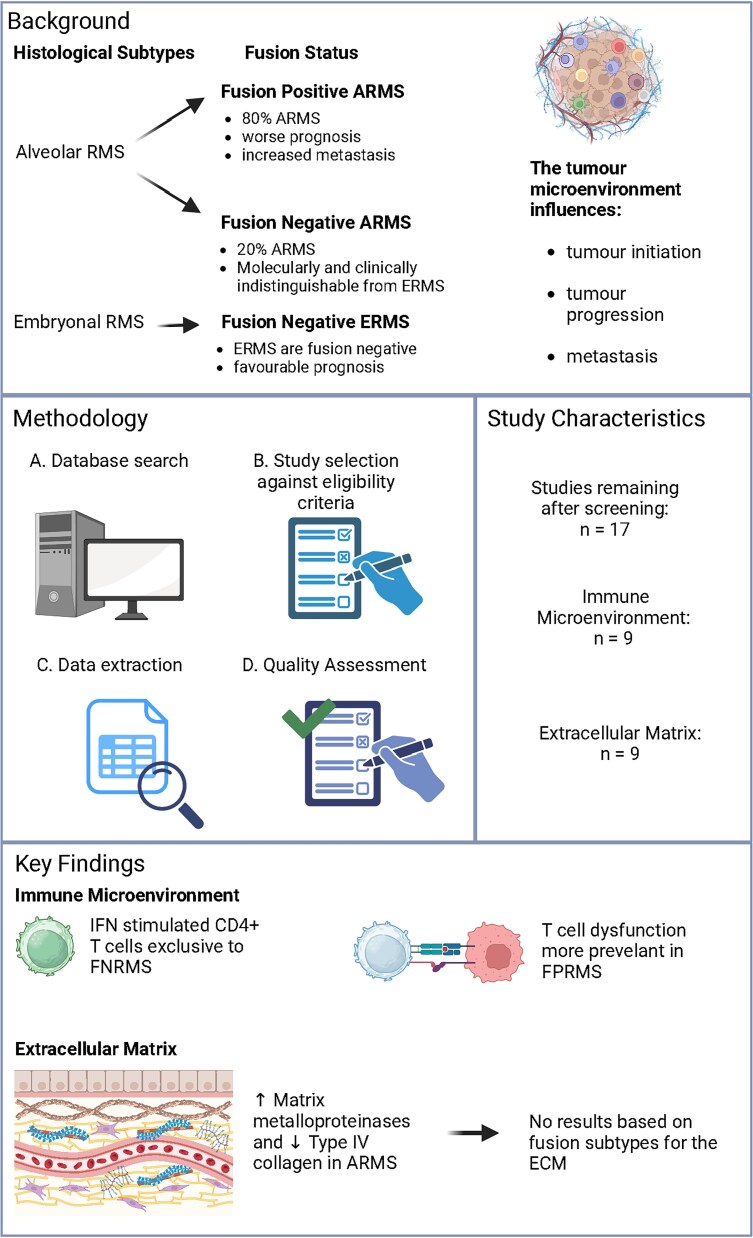

## 1. Introduction

### 1.1. Paediatric rhabdomyosarcoma

Rhabdomyosarcoma (RMS) is the most common type of soft tissue sarcoma (STS) in children, with an incidence of 4.3 cases per million a year worldwide [1]. Tumours express myogenic regulatory transcription factors, which suggest they are of myogenic origin and occur because of abnormal skeletal muscle differentiation [2–4].

Historically, RMS has been divided into alveolar, embryonal, pleomorphic and sclerosing subtypes, based on histological features. Embryonal (ERMS) and alveolar (ARMS) subtypes are the most common in children [4]. However, more recently, RMS has been defined by molecular characterization. Up to 80% of ARMS are fusion positive, characterized by a chromosomal translocation between *FOXO1* on chromosome 13 and either *PAX3* on chromosome 2 (t(2;13)(q35;q14) [5], or *PAX7* on chromosome 1 (t(1;13)(p36;q14) [6]. This results in the expression of more potent transcription factors PAX3-FOXO1 (in 55% of ARMS) or PAX7-FOXO1 (in 22% of ARMS) [7], leading to the classification of either fusion-positive RMS (FP-RMS) or fusion-negative RMS (FN-RMS). The presence of the *PAX-FOXO1* fusion is now known to have a primary role in RMS progression and drives an unfavourable outcome for children [8, 9]. FP-RMS have a significantly poorer overall survival, event free survival, and a higher frequency of metastasis compared with FN-RMS [8], making it more clinically aggressive. The fusion gene is also associated with disease recurrence, which decreases patient survival rate from 70%, after current multimodal therapy, to 20% [10, 11]. This highlights the need for novel and improved treatment strategies for FP-RMS patients.

Histology was traditionally used to predict prognosis, as the International Classification of Rhabdomyosarcoma identified ARMS as having a poorer prognosis compared with ERMS [9]. But recent evidence has shown that fusion-negative ARMS (FN-ARMS) is molecularly and clinically indistinguishable from ERMS [8]. Furthermore, fusion status has been shown to be a better predictor of prognosis in RMS and thus fusion status has now replaced histology in treatment stratification [9].

### 1.2. The tumour microenvironment

The Tumour Microenvironment (TME) plays a crucial role in tumour initiation, progression and metastasis [12, 13]. It consists of cancerous cells, as well as non-malignant host cells, including stromal cells (fibroblasts, endothelial cells, and pericytes), immune cells [CD8+ T cells, Natural Killer (NK) cells, Dendritic Cells (DCs), and macrophages] and the Extracellular Matrix (ECM); a non-cellular component, which comprises of a complex mixture of collagens, laminins, glycoproteins, and proteoglycans [14, 15].

Cancer cells communicate with the surrounding non-malignant components of the TME, stimulating changes in their function that promote tumour progression [15, 16]. The stroma is essential for maintaining the integrity of normal tissues; however, malignancy is associated with changes in the stroma, leading to tumour growth, invasion, and metastasis [17]. Furthermore, the immune microenvironment has a critical role in the surveillance and elimination of tumours; however, interactions between tumour cells and components of the immune TME can lead to immunosuppression and immune escape [18].

**Figure 1:**
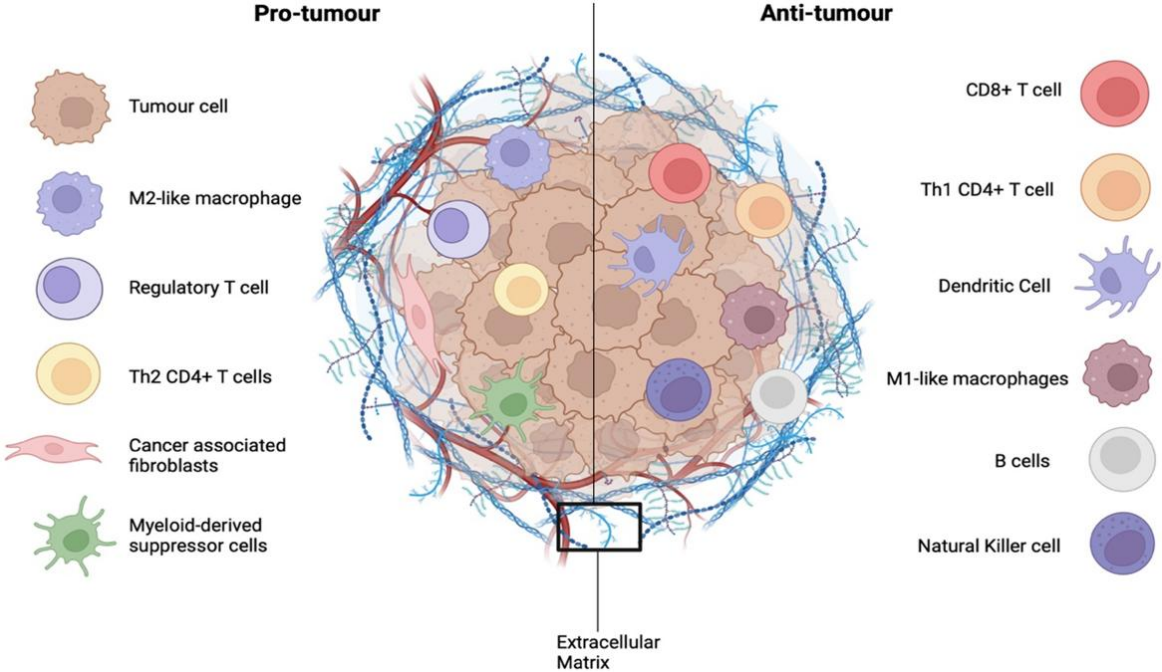
Pro-tumour (left) and anti-tumour (right) components of the tumour microenvironment. The tumour microenvironment (TME) is a complex ecosystem composed of stromal cells, immune cells, and a non-cellular component, known as the extracellular matrix. The TME can be split into pro-tumour components, which support tumour progression, and anti-tumour components, which function to inhibit tumour progression. (Created with BioRender.com, accessed on 14 September 2024.)

The immune TME can be grouped into pro-tumour and anti-tumour components (Fig. 1). Anti-tumour immune cells include CD8+ T cells, NK cells, DCs, and M1 polarized macrophages, which are associated with improved patient outcome in a variety of cancers [18-22]. Pro-tumour immune cells include regulatory T cells (T-regs), Myeloid-Derived Suppressor Cells (MDSCs), and M2 polarized macrophages, which are associated with tumour progression, invasion, and metastasis, resulting in poor patient prognosis [18, 23-28]. Advanced understanding of the TME has shifted the treatment of cancer, from direct targeting of tumour cells, to targeting the TME [12].

Previous studies have identified differences in the TME between cancer subtypes, which may shape their prognostic differences [13]. For example, HER2-positive breast cancer is associated with a higher immune score and immune infiltration compared with luminal A and luminal B subtypes [29]. The intensity of collagen type III staining has also been reported to vary between histological STS subtypes [30]. It was thus suggested that these differences in the TME between cancer subtypes may reflect their prognostic differences. In sarcomas, differences in the TME can be attributed to the presence of a fusion gene. For example, by comparing myxoid liposarcoma cells with or without the FUS::DDIT3 fusion protein, Ranji et al. [31] identified FUS::DDIT3 regulated genes involved in cell–cell and cell–ECM interactions, including those involved in ECM organization. There is a lack of understanding of how the presence of the PAX-FOXO1 fusion protein contributes to tumourigenesis and increased clinical aggression in the FP-RMS subtype [32]. Previous studies using genome wide screens have reported PAX-FOXO1 to exert oncogenic effects by the altered transcription of PAX3 target genes, which are involved in cell survival, myogenic differentiation, and mesodermal development [32, 33]. While these studies have attempted to identify these individual genes, many still require validation and further study for their role in FP-RMS progression [32]. In addition, these studies were also confounded by the inclusion of RMS with unknown fusion status [32]. It has also been concluded that PAX-FOXO1 alone is not sufficient to cause transformation [32]. Therefore, further work is required to precisely define the mechanisms underlying how the PAX-FOXO1 fusion proteins contribute to tumourigenesis. Since the TME has a crucial role in tumour progression, identifying differences between FP-RMS and FN-RMS may help to determine whether the TME contributes to more malignant behaviour in FP-RMS. This may in turn help identify novel targets for development of more effective treatment strategies for these FP-RMS patients.

### 1.3. Hypothesis, aims and objectives

This systematic review asked whether there were differences in the TME between paediatric FP-RMS and FN-RMS. Histological subtype was investigated to infer differences in the TME between fusion subtypes as histology has historically been used to classify RMS subtypes, rather than fusion status. Additionally, since the majority (around 80%) of ARMS are fusion positive, analysing TME differences between ERMS (which are fusion negative) and ARMS, could offer surrogate insights into differences between FP-RMS and FN-RMS.

The aim of this systematic review was to identify and evaluate current literature investigating the TME in patient-derived, paediatric FP-RMS and FN-RMS/ARMS and ERMS, to identify any differences in the stromal, immune, and ECM components between FP-RMS and FN-RMS. Since the TME influences tumour aggression [34], we hypothesized that, after synthesizing existing data from all available relevant studies of patient-derived samples, there would be a difference in the expression, proportion, number, type, and composition of immune, stromal, and ECM components between paediatric FN-RMS and the more clinically aggressive FP-RMS.

## 2. Methods

### 2.1. Search strategy

A systematic search of the literature (between 1994 and 2023) was conducted on three main databases: The Web of Science, MEDLINE (Ovid), and EMBASE (Ovid), with the help of a librarian at the University of Southampton. The Web of Science was searched using only free text terms, whereas EMBASE and MEDLINE were searched using both free text terms and subject heading terms. The search terms used across each database is presented in Table 1. Searches were limited to English language publications across all databases. In the Web of Science, searches were additionally restricted to published articles and in EMBASE, pre-print records were removed. This systematic review followed the recommendations of the Preferred Reporting Items for Systematic Reviews and Meta-Analysis (PRISMA).

**Table 1:** Search strategy used for the systematic search of the literature.

Database (platform)	Search strategy
Web of Science	(microenvironment OR matrix OR niche OR “tum*r microenvironment” OR “immune cell$” OR stroma OR “extracellular matrix” OR “cancer associated fibroblast$” OR “tum*r infiltrating lymphocyte$” OR “immune microenvironment” OR immune OR “tum*r immunology”) AND (rhabdomyosarcoma*) AND (paediatric* OR pediatric* OR child* OR infant* OR adolescen*)
MEDLINE (Ovid)	microenvironment or matrix or niche or “tumo?r microenvironment” or “immune cell$” or stroma or “extracellular matrix” or “cancer associated fibroblast$” or “tumo?r infiltrating lymphocyte$” or “immune microenvironment” or immune or “tumo?r immunology” or tumor microenvironment/or fibroblasts/or cancer-associated fibroblasts/or myofibroblasts/or macrophages/or tumor-associated macrophages/or stromal cells/or extracellular matrix/or cancer-associated fibroblasts/or -lymphocytes/or lymphocytes, tumor-infiltrating/or immune evasion/or immune checkpoint inhibitors/or immune system/or immunotherapy/) AND (rhabdomyosarcoma* or exp rhabdomyosarcoma/) AND (paediatric* or pediatric* or child* or infant* or adolescen*.mp. or infant/or child/or adolescent/or pediatrics/)
EMBASE (Ovid)	(microenvironment or matrix or niche or “tumo?r microenvironment” or “immune cell$” or stroma or “extracellular matrix” or “cancer associated fibroblast$” or “tumo?r infiltrating lymphocyte$” or “immune microenvironment” or immune or “tumo?r immunology” or tumor microenvironment/or exp stroma cell/or stroma cell/or bone marrow stroma cell/or stroma/or mesenchymal stroma cell/or extracellular matrix/or cancer associated fibroblast/or exp tumor associated leukocyte/or tumor microenvironment/or immune evasion/or immune system/or immune dysregulation/or immune signaling/or immune response/or immune checkpoint inhibitor/or exp tumor immunology/) AND (rhabdomyosarcoma* or rhabdomyosarcoma/or alveolar rhabdomyosarcoma/or embryonal rhabdomyosarcoma/) AND (paediatric* or pediatric* or child* or infant* or adolescen* or pediatrics/or child/or hospitalized child/or child hospitalization/or adolescent disease/or adolescent/or hospitalized adolescent/)

### 2.2. Inclusion and exclusion criteria

Inclusion and exclusion criteria were developed based on the PICO framework (Table 2). Studies were included if they met the following criteria: (i) reported patient-derived data from paediatric ARMS or ERMS/FP-RMS or FN-RMS (patient-derived data included patient-derived cell culture, patient-derived organoids, and patient-derived tissue slice cultures) and (ii) characterised a component(s) of the TME. Histological subtype and fusion subtype were included, as many studies still rely on histology to classify RMS. Paediatric was defined as children and young adults up to the age of 21.

**Table 2:** PICO framework and inclusion and exclusion criteria used for screening.

PICO framework		Inclusion criteria	Exclusion criteria
Population	Paediatric^a^ RMS	Paediatric ARMS or ERMS/FP-RMS or FN-RMS patient-derived data which includes: Patient-derived cell culture Patient-derived organoids Patient-derived tissue slice cultures	Adult RMS Animal and cell line models Patient-derived xenografts Studies that don’t separate RMS results from other sarcoma results. If study doesn’t specify whether the samples are from paediatric or adult patients, exclude at full text screening stage.
Intervention	The TME	Data that characterises a component(s) of the TME	Studies that do not describe the TME
Comparator	The TME in FP-RMS versus FN-RMS or ERMS versus ARMS.	Data from either ARMS or ERMS/FP-RMS FN-RMS	Studies that do not specify the subtype of RMS to be excluded only at the full text screening stage. Studies that group results from multiple subtypes together, to be excluded at the full text stage only. Pleomorphic or sclerosing subtypes.
Outcome	Differences in the TME between subtypes.	Expression, number, proportion, type and composition of immune, stromal and ECM components of the TME	
Other			Meeting abstracts Reviews Not full text articles

^a^Paediatric includes children and young adults up to the age of 21.

RMS, rhabdomyosarcoma; TME, tumour microenvironment; FP-RMS, fusion-positive rhabdomyosarcoma; FN-RMS, fusion-negative rhabdomyosarcoma; ARMS, alveolar rhabdomyosarcoma; ERMS, embryonal rhabdomyosarcoma.

Studies were excluded if they: (i) reported data from adult RMS; (ii) reported data from animal models, cell lines, or patient-derived xenograft models; (iii) did not characterize a component of the TME (or were not relevant to the TME); and (iv) reported data from pleomorphic or sclerosing subtypes. If studies did not specify the subtype of RMS, or whether samples were paediatric or adult in their abstracts, they were retained at the abstract screening stage. If not specified in their full text, they were excluded. Adult RMS was excluded, as it is much rarer and has a different prognosis compared with its paediatric counterparts [35].

### 2.3. The selection process

References identified from the search were exported to Rayyan (http://rayyan.qcri.org/) and de-duplicated. Rayyan was used to screen all titles and abstracts against the inclusion and exclusion criteria as stated in Table 2, to identify those that were potentially relevant. A random selection of 22 titles and abstracts were independently screened by a second reviewer. Any discrepancies were discussed and resolved. Full text papers were obtained using Endnote or manually searched for using the Web of Science, MEDLINE (Ovid), or EMBASE (Ovid). Some were not obtained due to the University not having access. Full texts were further screened against the inclusion and exclusion criteria to assess their relevance.

### 2.4. Data extraction

Data extracted from the included studies included: (i) first author and year; (ii) study aims; (iii) tissue processing technique; (iv) histological subtype and the number of samples of each; (v) fusion status and the number of samples of each; (vi) patient age; (vii) the experimental method(s) used; (viii) the component(s) of the TME analysed; and (ix) the key findings. In Chen et al.’s [36] study, key findings regarding their ARMS samples were not extracted, as not all samples were paediatric. Data extraction was conducted by one independent reviewer. Due to the heterogeneity in the methods used and the results reported among the studies, a meta-analysis could not be conducted, so the results were reported descriptively.

### 2.5. Quality assessment

To assess the quality of each included study, the Risk of Bias in Non-randomized Studies of Interventions (ROBINS-I) tool was used, which included seven domains of bias: confounding bias, selection bias, information bias, performance bias, detection bias, reporting bias, and bias due to missing data. The Cochrane Online Handbook for Systematic Reviews [37] was also used to help further clarify bias definitions. Studies were scored with either a low, moderate, serious, or critical risk of bias. Results of the quality assessment are presented in Supplementary Table S1.

## 3. Results

### 3.1. Study selection

**Figure 2:**
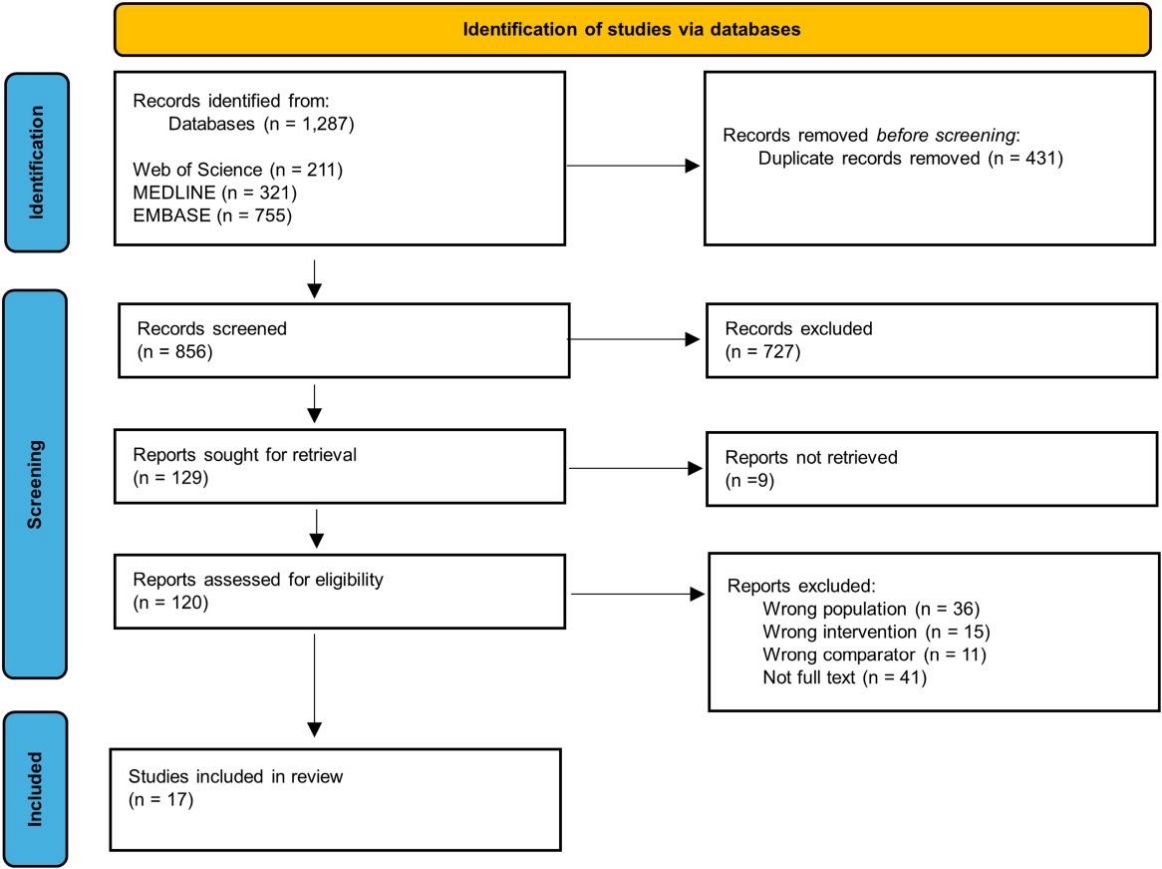
PRISMA flowchart of the process of article selection, using Preferred Reporting Items for Systematic Reviews and Meta-Analysis (PRISMA) guidelines.

The initial database search identified 1287 references in line with the initial search strategy. Of these, 211 were identified from the Web of Science, 321 from MEDLINE (Ovid), and 755 from EMBASE (Ovid). Following exportation to Rayyan, 431 duplicate records were removed, yielding a total of 856 references to be screened. The titles and abstracts were screened against the inclusion and exclusion criteria, which resulted in the exclusion of 727 references. Of the remaining 129, nine full text papers could not be retrieved, leaving 120 full texts to which inclusion criteria was applied. Full text screening resulted in 103 references being excluded. Full texts were excluded due to not meeting either the population criteria (n = 36), the intervention criteria (n = 15), the comparator criteria (n = 11), or were not full text articles (n = 41). Following study selection, 17 studies were included in this systematic review. The PRISMA flowchart of this selection process is presented in Fig. 2. The study characteristics of these included studies are summarized in Table 3 and the key findings are summarized in Table 4.

**Table 3:** Key characteristics of the studies included in the review.

First author, year	Reference	Study aims	Tissue processing technique	Histological subtype (number of samples)	Fusion status (number of samples)	Age of patients	Methods used for analysis	Component(s) of TME investigated
Chen et al. (2020)	[36]	Perform an in-depth interrogation of the tumour immune microenvironment of STS to identify immunotherapy agents.	FFPE tumour tissue	ERMS (27)	FP (50) FN (63)	ERMS: range = 0.02–22 mean = 7	IHC RNA sequencing gene expression analysis	Immune microenvironment
Bertolini et al. (2018)	[38]	Further describe the immune microenvironment of RMS by evaluating PD-L1 expression.	FFPE RMS tissue	ERMS (13) ARMS (11)	FP (7) FN (18)	N/A	IHC	Immune microenvironment PD-L1
Xu et al. (2022)	[39]	Delineate the testicular ERMS intra-tumoural heterogeneity and TME. Analyse the role of each subpopulation of cells in the progression of testicular ERMS. Analyse the relationship between macrophages and ERMS tumour cells.	Fresh tumour tissue	ERMS (1)	N/A	16 years	Single cell RNA sequencing	Macrophages Myoid cells Endothelial cells Fibroblasts
Xia et al. (2021)	[40]	Evaluate potential indicators of TME status changes. Correlate immune infiltration with low and high expression of MD2L1 and CCNB2 expressing populations in RMS samples.	Paraffin embedded RMS samples	ERMS (14) ARMS (12)	N/A	Study from which the dataset was from: mean = 7 range = 0–20	Bioinformatics IHC	Immune infiltration MD2L1 CCNB2
Saxon et al. (1997)	[41]	Investigate the presence of adhesion factors laminin, fibronectin, tenascin, thrombospondin and CD44 in paediatric RMS	Paraffin embedded samples	ERMS (6) ARMS (5)	N/A	ARMS: average = 11.6 years. Range = 6–15.4 years. ERMS: average = 6 years range = 2.4–8.2 years	IHC	Adhesion molecules: Laminin Fibronectin Tenascin Thrombospondin CD44
Gabrych et al. (2019)	[42]	Assess PD-L1 and PD-1 expression in paediatric RMS and to investigate their clinicopathological associations.	Biopsy samples	ERMS (19) ARMS (12)	N/A	Range = 1 day-18 years. Median = 7.4 years	IHC	PD-L1 and PD-1 expression
Vela et al. (2019)	[43]	Analysed CXCR4 expression in tumour samples from paediatric RMS patients. Used an orthotopic model of ARMS to evaluate the complementary antitumoural and antimetastatic effects of combined NKAE + MDX1338 immunotherapy *in vivo*.	FFPE tumour samples	ERMS (15) ARMS (5)	N/A	ERMS: mean = 5.1 range = 0.2–13 ARMS: mean = 9.8 range = 7.5–11.1	IHC	CXCR4 expression
Masola et al. (2009)	[44]	Analyse HPSE expression and activity in ARMS and ERMS and investigate the relationship of the different metastatic phenotypes of ARMS and ERMS with expression and activity of HPSE.	Plasma and tumour RNA collected from RMS patients	ERMS (10) ARMS (5)	N/A	Mean = 6.3 years Range = 1–15 years	HPSE mRNA expression was evaluated by real time PCR. HPSE activity determined by ELISA method. Plasma assay	HPSE
Peng et al. (2015)	[45]	N/A	N/A	ERMS (N/A) ARMS (N/A)	N/A	N/A	Morphology and number of TAMs examined by IHC.	TAMs APN
DeMartino et al. (2023)	[4]	Compile a single cell transcriptomic atlas comprising both FP-RMS and FN-RMS and to find distinct differences in cellular composition and differentiation states which relate to clinical outcomes.	Viably frozen primary RMS tumour samples Patient-derived tumour organoid models	N/A	FP (13) FN (13)	15 samples were <10 years 12 samples were >10 years	Single cell mRNA sequencing	Immune microenvironment
Martin et al. (2007)	[46]	Analyse dystroglycan expression and glycosylation in paediatric solid tumours and demonstrate alterations in α-dystrogycan in paediatric RMS (and others).	Snap frozen unfixed tumour samples	Immunoblot: ERMS (2) ARMS (1) Immunostaining: ERMS (23) ARMS (14)	N/A	Table 1: ERMS: mean = 4.5 years (5 + 4) ARMS: age = 9	Immunoblot for dystroglycan expression. Immunostaining on tissue microarrays.	Glycosylated dystroglycan
Thakur et al. (2022)	[47]	Investigate the immune microenvironment of five major paediatric cancers: Ewing sarcoma, osteosarcoma, RMS, medulloblastoma, neuroblastoma, and correlated with overall survival.	FFPE sections	ERMS (N/A) ARMS (N/A)	N/A	0–17	Gene expression analysis IHC	Immune microenvironment
Strahm et al. (2008)	[48]	To investigate the role of the bone marrow microenvironment on RMS signalling and behaviour through the CXCR4/SDF-1α pathway.	Patient RNA samples	ERMS (7) ARMS (5)	N/A	N/A	RT-PCR	Bone marrow stroma CXCR4/SDF-1α metastatic signalling
Stracca-Pansa et al. (1994)	[49]	Understand ECM elements in small round cell tumour tissue and to investigate the detection of these elements by IHC on tumours from patients.	FFPE tissue	ERMS (15) ARMS (9)	N/A	Range = 0.5–23 Mean = 7.5	IHC	ECM: Laminin Type IV collagen Fibronectin
Ehnman et al. (2013)	[50]	Identify biological activities linked to PDGF signalling in RMS models and human sample collections.	N/A	ERMS (261) ARMS (50)	N/A		Microarray analysis on 3 x tissue microarrays	PDGFRα and PDGFRβ
Diomedi-camassei et al. (2004)	[51]	To assess the expression of MMPs in RMS and to evaluate the correlation with clinicopathologic parameters.	FFPE tissue	ERMS (21) ARMS (12)	N/A	Mean = 85+/− 54 months Range = 2–175 months	IHC	MMPs
Chowdhury et al. (2015)	[52]	Report the frequency of PD-L1 expression in paediatric malignancies and to assess the frequency of tumour infiltrating CD8+ cytotoxic T-lymphocytes and their levels of PD-1 expression.	FFPE primary tumour samples	ERMS (18) ARMS (15)	N/A	ERMS: median = 6.5 range = 1.3–16.2 ARMS: median = 6.0 range = 1.2–12.8	IHC	PD-1/PD-L1 expression CD8+ cytotoxic T cells

FFPE, formalin-fixed paraffin embedded; IHC, immunohistochemistry; N/A, data not provided; TME, tumour microenvironment; RMS, rhabdomyosarcoma; ARMS, alveolar rhabdomyosarcoma; ERMS, embryonal rhabdomyosarcoma; ELISA, enzyme-linked immunosorbent assay; TAM, tumour associated macrophages; ECM, extracellular matrix; MMP, matrix metalloproteinase; FP, fusion positive rhabdomyosarcoma; FN, fusion negative rhabdomyosarcoma; STS, soft tissue sarcoma; RT-PCR, reverse transcription polymerase chain reaction; PDGF, platelet-derived growth factor; PDGFRα/β, platelet-derived growth factor receptor-α/β.

**Table 4:** Key findings of the studies included in the review.

Reference	Key findings
[36]	ERMS CD3 + and TAMs (CD163+) in close proximity to endothelial cells—majority of T cells and TAMs are found within 20–40 um of endothelial cells. TAMs predominated the immune microenvironment in all sarcomas. Majority of CD3 + and CD8+ T cells in aggregates with B cells forming TLS, which were the main source of PD-L1 expression. B cells found in TLS and not elsewhere. The common predominant immune signature was myeloid cells in RMS. Results from their analysis of publicly available gene expression datasets: There was a significantly higher number of resting CD4 + memory T cells in FN-RMS compared with FP-RMS (0.256+/−0.134 in FN-RMS compared with 0.185+/−0.118 in FP-RMS).
[38]	PD-L1 expression was heterogenous (not present at all in tumour cells) in both ARMS and ERMS. PD-L1 expression was found in the immune contexture in: 6/11 ARMS 9/14 ERMS. PD-L1 staining does not correlate with fusion status. PD-L1 expression colocalised with CD3+ T lymphocytes and CD68+ macrophages.
[39]	ERMS Myeoid cells showed downregulation of genes associated with ECM organization compared with the ERMS tumour cells (differentially expressed genes between tumour and normal were closely related to cell adhesion and ECM signalling pathways). Tumour cells included M1 and M2 macrophages, normal tissue contained M3. All macrophages (M1, M2 and M3) expressed immune checkpoint molecules, however M2 had a higher immune activity. M1 = FGL1 and IDO1 M2 = CD101 HAVCR2. M3 = CD86 E1 (34.41%), E2 (29.96%) and E3 (35.63%) endothelial cell subtypes expressed in tumour cells, normal tissues mainly expressed E2 (66.7%). E1 and E2 subgroups transform into E3 subgroup during progression to promote tumour progression. Macrophages were closely related to collagen and ECM pathway.
[40]	Bioinformatics analysis: ERMS: 7 showed low MAD2L1 expression (50%) and 7 showed high expression (50%). 5 showed low CCNB2 expression (35.7%) and 9 showed high expression (64.3%). ARMS: 3 showed low MD2L1 expression (25%) and 9 showed high expression (75%). 5 showed low CCNB2 expression (41.7%) and 7 showed high expression (58.3%). IHC analysis: Expression rate of MAD2L1 in RMS samples was 90.9% (30/33)—no expression in 11 control skeletal muscle tissue. Expression rate of CCNB2 was 100% (33/33)—no expression in control. No statistical difference in expression of MD2L1 or CCNB2 expression between ERMS and ARMS.
[41]	All samples expressed tenascin and thrombospondin regardless of subtype (5/5 ARMS, 6/6 ERMS). Fibronectin was expressed by all ARMS (5/5). Fibronectin not expressed in all ERMS (4/6). Laminin expressed in 3/5 ARMS and 2/6 ERMS. CD44 was not expressed by any ARMS but was expressed by half of RMS (3/6).
[42]	The positive PD-L1 staining in RMS samples was restricted to tumour associated immune cells in all cases, with no reactivity in tumour cells. No correlation between PD-L1 expression and RMS histological subtype (*P* = 0.451). ERMS PD-L1 + in 11/19 (57.89%). ARMS PD-L1 + 9/12 ARMS (75.00%).
[43]	No difference in CXCR4 expression between ERMS and ARMS (for diagnostic samples) (*P* = 0.59).
[44]	Plasma from ARMS patients showed higher activity levels of HPSE compared with ERMS but not statistically significant (data not shown). HPSE mRNA expression was significantly higher in both ERMS and ARMS compared with control.
[45]	CD163+ TAMs and APN + expressed at much higher levels in ARMS compared with ERMS (TAMs *P* < 0.05, APN *P* < 0.001)
[4]	The proportion of each non-malignant cell types did not differ significantly by fusion status. Non-malignant cell types included T cells (CD4 T cells, T-regs, CD8+ T cells), NK cells, B cells, monocytes and macrophages (M1 and M2). These non-malignant cell types were grouped into clusters—T/NK cell cluster, myeloid cluster, B cell cluster and endothelial cell cluster, whose proportions didn’t differ based on fusion status. IFN stimulated CD4+ T helper cells (ISG+) were found almost exclusively in FN-RMS. Dysfunction of CD8+ T cells was more prevalent in FP-RMS samples—CD8+ T cells were enriched for genes related to PD-1 signalling, OXPHOS and T cell exhaustion. Cells from FN-RMS tumours were enriched for gene signatures relating to IFN response and stimulation. Interaction between NECTIN3 on malignant cells and TIGIT receptor on T-regs and CD8+ T cells was specific to FP-RMS tumours due to significantly higher expression of NECTIN-3 on FP-RMS, whilst there was no difference in expression of TIGIT on CD8+ T cells between subtypes
[46]	Immunoblot results: All 5 RMS samples had reduced or absent expression of native α-dystroglycan. β-dystroglycan present in all RMS samples, molecular weight in tumour sample was similar to normal muscle controls. Laminin binding was significantly reduced in all 5 RMS samples compared with controls. Immunostaining of tissue microarrays results: Both ERMS and ARMS had significant reduction in α-dystroglycan staining relative to normal skeletal muscle and relative to β-dystroglycan. Laminin expression was significantly reduced relative to β-dystroglycan in both RMS subtypes. α-dystroglycan staining occurred primarily in tumour vasculature in both ERMS and ARMS. β-dystroglycan staining was abundant in all tumour samples of both tumour types—no tumour types received a score of 1 (no staining) for β-dysrtroglycan). For α-dystroglycan, 67% (for VIA4–1) and 55% (for IIH6) ERMS had a score of 1, and 43% (for VIA4-1) and 33% (for IIH6) of ARMS had a score of 1. ERMS (23): * between tumour sample and control βDG = 2.91 ± 0.21 VIA4-1 (αDG) = 1.46 ± 0.11*** IIH6 (αDG) = 1.70 ± 0.15*** LN-1 (laminin) = 1.66 ± 0.21* ARMS (14): * between tumour sample and control βDG = 2.89 ± 0.07 VIA4-1 (αDG) = 1.55 ± 0.16** IIH6 (αDG) = 1.90 ± 0.19* LN-1 (laminin) = 1.80 ± 0.13*
[47]	CD8 by IHC and gene expression was comparable in ARMS and ERMS. 30% of RMS samples were positive for PD-L1–4 samples had PD-L1 positivity in tumour cells, 2 samples were PD-L1 positive in immune cells.
[48]	CXCR4 expression was statistically increased in ARMS primary samples relative to ERMS and control skeletal muscle (*P* = 0.004). No statistical difference in expression of CXCR4 between ERMS and control skeletal muscle. All BMS cultures expressed increased SDF-1α. CXCR4- SDF-1α signalling axis may be involved in RMS metastasis to bone marrow.
[49]	ERMS: Laminin (93%) Fibronectin (87%) Type IV collagen (54%) ARMS: rarely expressed any of the extracellular matrix proteins Laminin (22%) Fibronectin (22%) Type IV collagen (14%)
[50]	PDGFRβ was significantly over expressed in both ARMS and ERMS. PDGFRβ protein levels were rarely detected in tumour cells, expression was associated with stroma in ARMS subtype. PDGFRβ stromal staining was positively associated with ARMS (*P* < 0.0001). PDGFRα expression was observed in both the tumour cell and stroma and was associated with ERMS. PDGFRα stromal staining was positively associated with ERMS (*P* = 0.0329).
[51]	ARMS cells stained diffusely and strongly for MMP-2 and MMP-9. MMP-1 positive in: 11/12 ARMS 11/21 ERMS MMP-2 positive in: 12/12 ARMS 9/21 ERMS MMP7 expressed in 26/33 tumour samples: 11/12 ARMS 15/21 ERMS MMP-3 was negative in almost all RMS samples (28/32). MMP-3 positive in: 2/12 ARMS 3/21 ERMS MMP-9 was positive in perivascular ECM and in vascular structures of tumours Positive in: 12/12 ARMS 11/21 ERMS Statistically significant difference between ARMS and ERMS: MMP-1 (*P* = 0.006) MMP-2 (*P* = 0.0001) MMP-9 (*P* = 0.0001)
[52]	86% of ARMS were PD-L1 positive. 50% of ERMS were PD-L1 positive. ERMS have a higher mean total number of CD8+ TILs compared with ARMS (significance wasn’t reported).

TAM, tumour associated macrophages; TLS, tertiary lymphoid structure; RMS, rhabdomyosarcoma; ERMS, embryonal rhabdomyosarcoma; ARMS, alveolar rhabdomyosarcoma; FN, fusion negative; FP, fusion positive; ECM, extracellular matrix; APN, adiponectin; NK cells, natural killer cells; IHC, immunohistochemistry; BMS, bone marrow stroma; FN-RMS, fusion negative rhabdomyosarcoma; FP-RMS, fusion positive rhabdomyosarcoma; OXPHOS, oxidative phosphorylation; T-regs, regulatory T cells; MMP, matrix metalloproteinase; TIL, tumour infiltrating lymphocytes; PDGFRα/β, platelet-derived growth factor receptor-α/β; SDF-1, stromal cell-derived factor 1; βDG, β-dystroglycan; αDG, α-dystroglycan.

### 3.2. Study characteristics

The 17 included studies were published between 1994 and 2023. Of these studies, only three included fusion status [4, 36, 38], the rest included only histological subtype (ERMS and ARMS). Nine studies investigated the ECM and the stroma [39, 41, 43, 44, 46, 48-51], another nine investigated the immune microenvironment [4, 36, 38-40, 42, 45, 47, 52], and one study investigated both aspects [39]. The most frequently used sample type was formalin-fixed, paraffin-embedded tissue, which was used by just over half of the studies (56%) [36, 38, 40, 41, 43, 47, 49, 51, 52]. Only one study used patient-derived organoids [4]. Data from patient-derived samples, rather than cell lines or animal models, were included for this review, as these better recapitulate the heterogeneity, complexity, and pathophysiology of patient tumours and their TMEs [53]. A variety of different methods were used for analysis, with immunohistochemistry being the most common (used in 64.7% of studies) [36, 38, 40-43, 45, 47, 49, 51, 52]. The mean number and the total number of samples per histological subtype and fusion subtype is presented in Table 5. Three studies were excluded from the mean calculation for ERMS due to missing sample size data [4, 45, 47]. Five studies were excluded from the mean calculation for ARMS due to three having missing sample size data [4, 45, 47], and two studies not including ARMS samples [36, 39].

**Table 5:** The total number and the mean number of samples for each RMS subtype.

Subtype	Mean	Total
FP-RMS	23	70
FN-RMS	31	94
ERMS	32	452
ARMS	13	156

FP-RMS, fusion positive rhabdomyosarcoma; FN-RMS, fusion negative rhabdomyosarcoma; ERMS, embryonal rhabdomyosarcoma; ARMS, alveolar rhabdomyosarcoma.

### 3.3. Study results

#### 3.3.1. The immune microenvironment

Of the studies investigating the immune microenvironment, macrophages (n = 5) [36, 38-40, 45] and CD8+ T cells (n = 4) [4, 36, 47] were investigated the most frequently. Other immune components were also investigated, such as B cells [36], CD4+ T cells [4, 36], monocytes [4], NK cells [4], DCs [4], and T-regs [4]. Three studies investigated the immune microenvironment in FP-RMS and FN-RMS [4, 36, 38]. Data across studies could not be averaged due to heterogeneity in their methods and results reported.

Tumour-associated macrophages (TAMS), particularly M2-like TAMs, are an immunosuppressive immune cell of the TME [54]. Two studies investigated the difference in macrophages between the histological subtypes and fusion subtypes [4, 45]. DeMartino et al. [4] reported no difference in the proportion of macrophages between FP-RMS and FN-RMS, and reported that they existed predominantly in the M2 polarization state in both fusion subtypes. In contrast, Peng et al. [45] identified a difference in the expression of CD163, a marker specific for M2 macrophages, between ERMS and ARMS, with expression levels being significantly increased in ARMS. The latter study did not provide information regarding fusion status.

Cytotoxic CD8+ T cells are the primary effector cells of the anti-tumour immune response [55]. Thakur et al. [47] characterized CD8 expression and reported no significant difference between ERMS and ARMS. However, results presented by Chowdhury et al. [52] show a higher mean total CD8+ tumour infiltrating lymphocyte (TIL) count in ERMS compared with ARMS, although the significance was not reported. DeMartino et al. [4] reported that interferon (IFN) stimulated CD4+ T helper cells (ISG+) were found almost exclusively in FN-RMS tumours, which were enriched for gene signatures related to IFN response and stimulation. They also reported that T cell dysfunction was more prevalent in FP-RMS tumours, with CD8+ T cells enriched for genes related to PD-1 signalling and T cell exhaustion [4]. In addition, they reported NECTIN-3 to be upregulated on FP-RMS cells, whose interaction with TIGIT on T cells induces T cell dysfunction [56]. Chen et al. [36] found a difference in CD4+ memory T cells between fusion subtypes, with a significant increase in FN-RMS compared with FP-RMS. DeMartino et al. [4] also reported that the proportion of non-malignant cell types, which included T cells, NK cells, DCs, monocytes, and B cells, did not differ significantly based on fusion status.

The immune checkpoint molecules PD-L1, expressed on antigen-presenting cells and tumour cells, and PD-1, expressed on TILs, interact to inhibit T cell activation, which allows cancer cells to evade immunosurveillance [57]. Bertolini et al. [38] and Gabrych et al. [42] reported PD-L1 expression to be restricted to the immune contexture in their RMS samples, which had no correlation with histological subtype [42], or fusion status [38]. However, Chowdhury et al. [52] reported PD-L1 expression on the tumour cells, with 86% of ARMS being PD-L1 positive, compared with 50% of ERMS. But the statistical significance of this difference was not measured and information regarding fusion status was not provided.

#### 3.3.2. The extracellular matrix and the tumour stroma

Nine studies investigated a component of the ECM in RMS [39, 41, 43, 44, 46, 48-51], which included adhesion factors [41, 49], chemokine receptor 4 (CXCR4) signalling [43, 48], ECM enzymes [44], ECM receptors [46], matrix metalloproteinases (MMPs), [51] platelet-derived growth factor receptor (PDGFR) signalling [50], laminin and fibronectin [41, 49], and type IV collagen [49]. No studies investigated these components in FP-RMS or FN-RMS specifically.

Laminin and fibronectin are major proteins involved in establishing the architecture of the ECM [58]. Two studies investigated laminin and fibronectin expression in ERMS and ARMS and found opposing results [41, 49]. Saxon et al. [41] reported laminin and fibronectin to be expressed by a higher percentage of ARMS compared with ERMS, whereas Stracca-Pansa et al. [49] reported laminin and fibronectin to be expressed by a higher percentage of ERMS compared with ARMS. Again, the significance of these differences was not reported, and fusion status was not provided.

CXCR4 is commonly expressed on tumour cells [59]. Binding to its ligand, stromal cell-derived factor 1, in the TME, promotes angiogenesis, survival and proliferation of tumour cells, and recruitment of immune cells [60, 61]. Vela et al. [43] and Strahm et al. [48] investigated CXCR4 expression in ERMS and ARMS and found opposing results. Vela et al. [43] observed CXCR4 staining in 68% of their specimens but reported no difference in CXCR4 expression between ERMS and ARMS. However, Strahm et al. [48] observed a statistically significant increase in CXCR4 expression in ARMS compared with ERMS. Data from across these two studies could not be averaged due to differences in tissue processing techniques and experimental methods. Again, no fusion status was provided.

Heparanase, α- and β-dystroglycan, tenascin and thrombospondin, and type IV collagen are all ECM components, known to be important in cancer progression [62-65]. They were investigated by four studies using different methods [41, 44, 46, 49]. None of the studies reported information regarding fusion status. It was reported that there were no differences in the expression of heparanase [44], α- and β-dystroglycan [46], or tenascin and thrombospondin [41], between ERMS and ARMS. However, Martin et al. [46] reported that both subtypes had a significant reduction in α-dystroglycan and laminin binding relative to the control. Type IV collagen, an ECM protein, was expressed in only 14% of ARMS compared with 54% of ERMS [49] and CD44 was expressed in 50% of ERMS, but not at all in ARMS samples [41]. The significance of these results was not reported.

MMPs promote tumour cell invasion and metastasis by degrading the surrounding ECM [66]. Diomedi-Camassei et al. [51] reported a significant increase in the expression of MMP-1, MMP-2 and MMP-9 in ARMS compared with ERMS, but no difference in the expression of MMP-3 or MMP-7.

PDGFs and their receptors, PDGFRs, are expressed on tumour and stromal cells [67]. Ehnman et al. [50] reported a positive association between PDGFRβ stromal staining and ARMS, and a positive association between PDGFRα stromal staining and ERMS. Interestingly, they also showed that stromal PDGFRα was negatively associated with metastasis, whereas PDGFRβ was positively associated with metastasis, reflective of the more aggressive behaviour observed in ARMS.

## 4. Discussion

The composition of the TME plays a crucial role in supporting tumour survival and metastasis and has previously been shown to differ between aggressive and less aggressive cancer subtypes [15, 16]. The role of the PAX-FOXO1 fusion proteins in tumourigenesis lacks understanding, but FP-RMS is associated with a much more aggressive and metastatic nature compared with FN-RMS [8], which could be linked to differences in their TMEs. Therefore, the aim of this systematic review was to use systematic review methodology to identify and evaluate current literature investigating the TME in patient-derived, paediatric FP-RMS, FN-RMS/ERMS and ARMS in order to identify potential differences in the TME between the fusion subtypes.

This review identified significant differences in CD163+ macrophages [53], MMPs [45], and stromal PDGFRα/β [42] between ARMS and ERMS. An increase in T cell dysfunction and NECTIN-3 expression was reported in FP-RMS, with an increase in gene sets relating to PD-1 signalling and T cell exhaustion [4]. Genes relating to IFN response were enriched in FN-RMS samples [4]. There were no differences in the expression of α/β-dystroglycan, thrombospondin, tenascin, or heparanase expression between ERMS and ARMS [40, 46, 49, 50]. There were also no differences identified in the proportion of T cells, NK cells, myeloid cells, or B cells based on fusion status [4]. PD-L1 expression [43, 52, 55], CXCR4 expression [48, 51], laminin, and fibronectin [40, 46] expression were variable between studies.

### 4.1. The immune microenvironment

The immune microenvironment plays a crucial role in the elimination of tumours; however, tumours can evade the immune response by mechanisms such as immunosuppression and inducing T cell dysfunction [68]. TAMs are an immunosuppressive immune cell and have a direct role in the proliferation, invasion and metastasis of tumours [69]. Previous studies have shown that an increased infiltration of CD163+ TAMs, associated with an M2 phenotype, is associated with more advanced tumours with poorer prognosis [70].Consistent with the literature, this review found that the expression of CD163+ TAMs was significantly increased in ARMS compared with ERMS [45], suggestive of a more immunosuppressive TME in ARMS. This difference may be driven by the PAX-FOXO1 fusion protein, present in the ARMS subtype. DeMartino et al. [4] reported no difference in the proportion of macrophages based on fusion status [4].

Current literature has shown that cancer subtypes with more favourable prognosis are associated with a higher infiltration of CD8+ T cells [71]. In contrast to this, Thakur et al. [47] reported no significant difference in CD8 expression between ARMS and ERMS. But, since a small proportion of ARMS are fusion negative and are biologically and clinically indistinct from ERMS, these results could be confounded by the presence of FN-ARMS. This highlights the limitation of missing fusion status data.

Despite an infiltration of cytotoxic CD8+ T cells, tumours can evade the immune response by inducing T cell dysfunction [72]. T cell dysfunction was more prevalent in FP-RMS, and correlated with an enrichment of genes related to T cell exhaustion and PD-1 signalling, as well as an upregulation of NECTIN-3 on FP-RMS cells [4]. These results suggest a reduction in the activity of cytotoxic T cells in FP-RMS, which may be contributing to its poor prognosis. This is reflective of current knowledge that immune evasion is a crucial mechanism contributing to tumour progression and metastasis [73]. Similar to DeMatino et al.’s [4] study reporting an increase in PD-1 signalling in FP-RMS, Chowdhury et al.’s [52] study showed PD-L1 to be expressed by a higher percentage of ARMS cells compared with ERMS cells. This suggests an increase in PD-L1 expression associated with the presence of the fusion gene, potentially contributing to the aggressive and metastatic nature of FP-RMS. This correlates with previous research showing PD-L1 expression to be increased in metastatic tumours compared with primary tumours [74]. However, the significance of Chowdhury et al.’s [52] findings were not reported.

DeMartino et al.’s [4] study found that cells of FN-RMS tumours were enriched with IFN response and stimulation gene signatures, indicative of an IFN response in these tumours, which may be contributing to the better prognosis observed in FN-RMS patients. IFNs are potent drivers of the anti-tumour immune response and play a critical role in shaping the TME [75]. DeMartino et al. [4] identified that IFN stimulated CD4+ T helper cells (ISG+) were found almost exclusively in FN-RMS tumours, reflective of IFNs role in promoting a Th1 phenotype of CD4+ T cells [4, 75]. Future research should further investigate IFN activity and the resulting impacts it has on the immune cells of the TME between fusion subtypes. This could provide insight into how distinct immune responses contribute to differences in prognosis between FP-RMS and FN-RMS.

### 4.2. The extracellular matrix and the tumour stroma

Paediatric sarcomas have previously been characterized by high levels of MMPs, which are secreted by various cells of the TME to promote tumour cell migration, invasion, and eventually metastasis by degrading the surrounding ECM [76, 77]. Consistent with this, Diomedi-Camassei et al. [51] identified that MMP-1, MMP-2, and MMP-9 were significantly increased in ARMS compared with ERMS, which reflects previous evidence showing that PAX3-FOXO1 may promote a metastatic phenotype by increasing MMP-2 activity [33, 78]. Degradation of the ECM by MMPs involves the loss of basement membrane architecture, of which type IV collagen is a major component [79]. The reduced expression of type IV collagen in ARMS compared with ERMS [49] may therefore be reflective of the increase in MMPs in ARMS/FP-RMS. The interaction of these TME components may therefore be contributing to the more aggressive and metastatic nature of FP-RMS and should be an area of focus for future research.

Compared with the immune microenvironment, studies investigating the stromal component of the TME in RMS are lacking. The most abundant component of the tumour stroma are cancer-associated fibroblasts (CAFs), however these are largely understudied in many STSs due to a shared mesenchymal origin, making it challenging to distinguish CAFs from malignant mesenchymal cells [80]. However, the tumour stroma, including CAFs, has a role in promoting tumour development, metastasis, and recurrence, and has been correlated with phenotypes of tumour aggression in solid tumours [81]. Therefore, further understanding of the tumour stroma in paediatric RMS may provide valuable information regarding the mechanism of RMS progression, which could provide novel targets for treatment. Thus, future research should aim to overcome the challenges of investigating CAFs, to better characterize the tumour stroma in FP-RMS and FN-RMS.

### 4.3. Clinical significance

Like many paediatric solid tumours, RMSs are generally considered to be “immune cold”, owing to a low mutational burden and low/no neoantigen expression in tumours [82]. Our review highlights that, whilst there is a relative scarcity of information, RMS display low lymphoid infiltration, a predominance of M2-like, anti-inflammatory TAMs and limited CD8+ T cell infiltration, consistence with these tumours being overall immune cold [4, 36, 45, 47, 52]. However, there is limited evidence to suggest that FN-RMS/ERMS are somewhat more immune-permissive with higher CD8+ T-cell infiltration and fewer M2-like TAM [4, 36, 45, 52]. To date, there have been a limited number of clinical trials using immunotherapy approaches with reported outcomes. Approaches that have utilized checkpoint inhibitors (PD-1, PD-L1, CTLA4) showed very limited responses overall. Single agent nivolumab (PD-1) showed no responses in RMS patients [83], whereas nivolumab in combination with ipilimumab (CTLA-4) showed one partial response in RMS across a paediatric cohort [84]. The PD-L1 inhibitor atezolizumab in combination with chemotherapy showed a partial response in one RMS patient as part of a feasibility study [85] and efficacy is now being assessed in an on-going trial in RMS (NCT04796012).

More recently, focus has shifted to the potential for Chimeric Antigen Receptor (CAR) T and CAR Cytokine-Induced Killer (CIK) therapeutic approaches to treat RMS owing to high tumour cell surface antigen expression of key molecules including B7-H3, FGFR4 and GD2 [86-88]. A recent clinical trial using the monoclonal B7-H3 targeting antibody, enoblituzumab, showed no responses across paediatric patients including RMS [89]. However, preclinical evidence for CAR have shown promise with early-phase clinical trials in paediatric solid cancer patients currently undergoing assessment (NCT04483778). Additionally, clinical trials to assess CAR FGFR4 (NCT06865664) and CAR GD2 (NCT02107963) are currently underway and are likely to include RMS patients. However, given the low immune infiltration and immunosuppressive microenvironment seen in RMS tumours it remains to be seen whether these therapies will prove effective in the clinical setting.

### 4.4. Strengths and limitations

The methodology of this review allowed the aim of identifying and evaluating relevant literature to identify differences in the TME between FP-RMS and FN-RMS, to be achieved. The screening of a random selection of titles and abstracts by a second independent reviewer highlighted some discrepancies, which allowed clearer inclusion and exclusion criteria to be developed. For instance, it was decided that studies that did not specify the subtype, or whether samples were paediatric or adult in the abstract, were to be retained for screening of the full text. This ensured that all relevant studies were included. The data extraction was carried out by only one reviewer; however, the data that was extracted was double checked and additional information added during the quality assessment stage, which ensured that all relevant findings were included. In addition, a comprehensive search strategy was developed and translated appropriately between databases to ensure that all possible relevant literature were identified. Furthermore, multiple databases were used to search for literature, which is recommended to capture all relevant studies [90]. The three databases used [MEDLINE (Ovid), EMBASE (Ovid), and the Web of Science] were recommended by Barmer et al. [90] to be used for adequate recall, precision, and number of references. These steps ensured that all relevant literature were identified and included in the review.

However, there were also some notable limitations. Firstly, the majority of included studies had small sample sizes, attributed to the rarity of this cancer type. This meant that they lacked statistical power [91], which may explain the lack of difference identified in some TME components between subtypes. Some studies also failed to report if statistical significance was measured, making it difficult to draw conclusions based on their results. Smaller sample sizes also increase the chance of random variations in results [92], which could explain the contrasting results found between CXCR4 expression [43, 48], and laminin and fibronectin [41, 49] between studies.

This review looked at both histology and fusion status in order to identify differences in the TME between FP-RMS and FN-RMS. This approach was chosen as many studies still rely on RMS histology, which has historically been used to classify RMS. Additionally, since the majority of ARMS are fusion positive, and all ERMS are fusion negative, analysing differences in the TME between ERMS and ARMS could offer insights into differences between FP-RMS and FN-RMS. However, the absence of fusion status information in the included studies was a major limitation of this approach as it could not be determined whether differences in the TME between ERMS and ARMS were driven by the presence of the fusion gene in the ARMS samples. Absence of fusion status information could also contribute to the reporting of no difference in the TME between ERMS and ARMS, which could be confounded by the potential presence of FN-ARMS. Furthermore, a third subtype of RMS, known as spindle cell/sclerosing RMS (SS-RMS) has only recently been separated from the ERMS subtype, defined as a separate pathologic entity in the World Health Organisation (WHO) 2013 classification of soft tissue and bone tumours [93]. SS-RMS can be further classified by the presence of a *MYOD1* mutation, associated with a highly lethal outcome and unfavourable behaviour, which is comparable to ARMS [93]. Therefore, the possible presence of this RMS subtype in the ERMS samples, particularly in the older studies, may have contributed to a lack of difference in the TME reported between ERMS and ARMS.

This review also highlighted a lack of standardization of methods used to analyse some components of the TME, contributing to variability in results and preventing data from multiple studies being averaged. For instance, Bertolini et al. [38] and Gabrych et al. [42] detected PD-L1 expression only on tumour associated immune cells, while Chowdhury et al. [52] detected it on ARMS and ERMS tumour cells. Similar inconsistencies regarding PD-L1 expression have been found in previous research, which has been attributed to the use of different antibodies across studies [74]. Bertolini et al. [38] and Gabrych et al. [42] used SP142 antibody and 22C3 clone to detect PD-L1 expression, whereas Chowdhury et al. [52] used anti-CD274. To draw more reliable conclusions, more standardized methods should be used across studies.

## 5. Conclusion

This systematic review has highlighted that research regarding the TME in FP-RMS and FN-RMS is still in its infancy, with single-cell studies, such as DeMartino et al.’s [4] study, only beginning to highlight potential differences between these fusion subtypes. Because of this, there was insufficient evidence to draw robust conclusions about differences in the TME between FP-RMS and FN-RMS specifically. Findings based on histological subtypes suggest there may be differences linked to fusion status. But due to the fusion status being unknown in the majority of studies, further research is needed to confirm this. Future research should therefore prioritize investigating fusion subtypes over histological subtypes, since fusion status is now recognized to classify RMS and predict prognosis more accurately. It can also be recommended that an updated version of this systematic review be conducted as more data on the TME in FP-RMS and FN-RMS becomes available. Since the TME plays a crucial role in tumour progression, understanding its composition, in relation to fusion status, will improve our knowledge of how the *PAX-FOXO1* fusion gene contributes to tumourigenesis and a poorer prognosis seen in FP-RMS patients. Ultimately, this could identify novel targets and aid in the development of novel treatment strategies to improve patient outcomes.

## Supplementary Material

bgag011_Supplementary_Data

## Data Availability

No new data were generated or analysed in support of this research.
