# Supplementary material for: The tumour microenvironment in paediatric rhabdomyosarcomas: a systematic review
Source: Carcinogenesis. 2026 Feb 20;47(1):bgag011. doi: 10.1093/carcin/bgag011 (PMC13017844; doi:10.1093/carcin/bgag011)
Supplement: bgag011_Supplementary_Data [file bgag011_supplementary_data.docx]

**Supplementary Table S1** Assessment of the Risk of Bias of the included studies using the ROBINS-I tool

| **Reference** | **Were the confounding domains controlled for?** | | | | **Bias due to confounding** | **Bias in participant selection (selection bias)** | **Bias in classification of interventions (information bias)** | **Bias due to deviations from intended interventions (performance bias)** | **Bias due to missing data** | **Bias in measurement of outcomes (detection bias)** | **Bias in selection of reported result (reporting bias)** | **Risk of Bias** |
| --- | --- | --- | --- | --- | --- | --- | --- | --- | --- | --- | --- | --- |
|  | **Sample type** | **Treatment history** | **Tumour stage** | **Tumour origin** |  |  |  |  |  |  |  |  |
| [[39]](applewebdata://5AE95D2B-84C6-4312-99E6-09F86A4CA1B0/#ref39) | no | no | N/A | N/A | serious | N/A | N/A | N/A | low | low | serious | serious |
| [[41]](applewebdata://5AE95D2B-84C6-4312-99E6-09F86A4CA1B0/#ref41) | N/A | no | no | no | serious | N/A | N/A | N/A | low | low | serious | serious |
| [[42]](applewebdata://5AE95D2B-84C6-4312-99E6-09F86A4CA1B0/#ref42) | yes | yes | N/A | yes | low | critical | moderate | N/A | low | low | low | critical |
| [[51]](applewebdata://5AE95D2B-84C6-4312-99E6-09F86A4CA1B0/#ref51) | yes | yes | no | no | moderate | low | low | low | low | low | moderate | moderate |
| [[43]](applewebdata://5AE95D2B-84C6-4312-99E6-09F86A4CA1B0/#ref43) | yes | N/A | N/A | no | serious | serious | low | low | low | low | low | serious |
| [[52]](applewebdata://5AE95D2B-84C6-4312-99E6-09F86A4CA1B0/#ref52) | N/A | yes | no | no | moderate | Low | low | low | low | moderate | serious | serious |
| [[44]](applewebdata://5AE95D2B-84C6-4312-99E6-09F86A4CA1B0/#ref44) | yes | no | NI | NI | moderate | serious | low | low | moderate | moderate | serious | serious |
| [[45]](applewebdata://5AE95D2B-84C6-4312-99E6-09F86A4CA1B0/#ref45) | NI | NI | NI | NI | N/A | low | low | low | low | low | critical | critical |
| [[53]](applewebdata://5AE95D2B-84C6-4312-99E6-09F86A4CA1B0/#ref53) | Ni | Ni | Ni | Ni | NI | NI | NI | low | NI | low | serious | serious |
| [[4]](applewebdata://175E008F-7EF9-4579-AFD8-7B6DCD2AD6C3/#ref4) | no | no | no | no | critical | serious | low | moderate | low | low | serious | critical |
| [[46]](applewebdata://175E008F-7EF9-4579-AFD8-7B6DCD2AD6C3/#ref46) | NI | NI | NI | No | serious | low | low | low | low | low | low | serious |
| [[54]](applewebdata://175E008F-7EF9-4579-AFD8-7B6DCD2AD6C3/#ref54) | no | no | no | NI | serious | serious | low | low | low | low | low | serious |
| [[47]](applewebdata://175E008F-7EF9-4579-AFD8-7B6DCD2AD6C3/#ref47) | yes | Ni | Ni | Ni | Ni | Ni | moderate | low | low | low | serious | serious |
| [[48]](applewebdata://175E008F-7EF9-4579-AFD8-7B6DCD2AD6C3/#ref48) | Ni | Ni | Ni | Ni | NI | moderate | moderate | low | moderate | serious | moderate | serious |
| [[49]](applewebdata://175E008F-7EF9-4579-AFD8-7B6DCD2AD6C3/#ref49) | Ni | Ni | Ni | Ni | NI | critical | low | serious | serious | moderate | critical | critical |
| [[50]](applewebdata://175E008F-7EF9-4579-AFD8-7B6DCD2AD6C3/#ref50) | yes | yes | Ni | yes | Ni | low | low | low | low | low | low | low |
| [[55]](applewebdata://175E008F-7EF9-4579-AFD8-7B6DCD2AD6C3/#ref55) | yes | yes | Ni | Ni | N/A | low | low | low | low | low | serious | serious |

Ni, no information; N/A, not applicable
